# Reflex *BRCA1* and *BRCA2* tumour genetic testing for high-grade serous ovarian cancer: streamlined for clinicians but what do patients think?

**DOI:** 10.1186/s13053-022-00221-5

**Published:** 2022-04-13

**Authors:** Jeanna M. McCuaig, Sarah E. Ferguson, Danielle Vicus, Karen Ott, Tracy L. Stockley, Raymond H. Kim, Kelly A. Metcalfe

**Affiliations:** 1 University Health Network, 610 University Avenue, Toronto, M5G 2M9 Canada; 2 Lawrence S. Bloomberg Faculty of Nursing, University of Toronto, 155 College St, Toronto, M5T 1P8 Canada; 3 Familial Cancer Clinic – Princess Margaret Cancer Centre, 610 University Avenue, 700U-6W390, Toronto, ON M5G 2M9 Canada; 4 Department of Obstetrics and Gynecology, University of Toronto, 123 Edward Street, Toronto, M5G 1E2 Canada; 5 Sinai Health, 600 University Avenue, Toronto, M5G 1X5 Canada; 6 Sunnybrook Health Sciences Centre- Odette Cancer Centre, 2075 Bayview Avenue, Toronto, M4N 3M5 Canada; 7 Department of Laboratory Medicine and Pathobiology, University of Toronto, 1 King’s College Cir, Toronto, M5S 1A8 Canada; 8 Hospital for Sick Children, 555 University Ave, Toronto, M5G 1X8 Canada; 9 Department of Medicine, University of Toronto, 1 King’s College Cir, Toronto, M5S 1A8 Canada; 10 Adaptive Oncology, Ontario Institute for Cancer Research, 661 University Avenue, Toronto, M5G 0A3 Canada; 11 Women’s College Research Institute, 76 Grenville St., Toronto, M5G 1N8 Canada

**Keywords:** BRCA, Hereditary, Genetic testing, Genetic counselling, Somatic testing, Ovarian cancer, Psychological distress

## Abstract

**Background:**

Reflex (automatic) *BRCA1* and *BRCA2 (BRCA1/2)* genetic testing of tumour tissue is being completed for all newly diagnosed high-grade serous ovarian cancer (HGSOC) in the province of Ontario, Canada. The objective of this study was to measure the psychological impact of tumour genetic testing among individuals with a new diagnosis of HGSOC.

**Methods:**

Participants had a new diagnosis of HGSOC and received reflex *BRCA1/2* tumour genetic testing as a component of their care. Eligible individuals were recruited from two oncology centres in Toronto, Canada. One week after disclosure of tumour genetic test results, consenting participants were asked to complete a questionnaire that measured cancer-related distress, dispositional optimism, knowledge of hereditary breast/ovarian cancer, recall of tumour genetic test results, satisfaction, and the psychological impact of receiving tumour genetic test results. The Multidimensional Impact of Cancer Risk Assessment (MICRA) questionnaire was used to measure the psychological impact of tumour genetic testing.

**Results:**

76 individuals completed the study survey; 13 said they did not receive their tumour test results. Of the remaining 63 participants, the average MICRA score was 26.8 (SD = 16.3). Higher total MICRA scores were seen among those with children (*p* = 0.02), who received treatment with primary surgery (*p* = 0.02), and had higher reported cancer-related distress (*p* < 0.001). Higher dispositional optimism (*p* < 0.001) and increasing age (*p* = 0.03) were associated with lower total MICRA scores. Most (83.5%) participants reported being satisfied/highly satisfied with having tumour testing completed; however, 40.8% could not accurately recall their tumor test results.

**Conclusions:**

This study is the first to assess psychological outcomes following reflex *BRCA1/2* tumour genetic testing in women newly diagnosed with HGSOC. Increased dispositional optimism provided a protective effect, while increased cancer-related distress increased the psychological impact of tumour genetic testing. Educational resources are needed to help increase patient understanding and recall of tumour results, particularly when tumour genetic testing includes analysis of genes that may have implications for hereditary cancer risk. Additional research is required to better understand the patient experience of reflex tumour genetic testing.

## Introduction

An estimated 20–30% of high-grade serous ovarian cancers (HGSOC) are attributed to a pathogenic or likely pathogenic variant (PV), colloquially known as ‘mutation’, in a cancer-risk gene [1–3]. PVs can be inherited (germline) and passed from generation to generation, or can only be present in the tumour tissue (somatic), which has no implications for family members. The majority of PVs conferring ovarian cancer risk occur in the *BRCA1* or *BRCA2* genes (herein: *BRCA1/*2); however, additional ovarian cancer risk genes have also been identified [4].

For women with an inherited (germline) PV in an ovarian cancer risk gene, there is a 50% chance of passing the risk on to each of their children. While screening for ovarian cancer has not been shown to improve outcomes [5], risk-reducing bilateral salpingo-oophorectomy has been associated with an 70–96% reduction in ovarian cancer risk and a 60–77% reduction in all cause mortality among women with a PV [6–8]. Thus, genetic testing to identify a hereditary cancer risk is recommended for all HGSOC, irrespective of age at diagnosis or family history [9].

Recent advances in the treatment of ovarian cancer, has prompted the widespread use of genetic testing for women with HGSOC. Treatment of ovarian cancer with poly(ADP-ribose) polymerase inhibitors (PARPi) has resulted in improved survival for individuals with a germline or somatic PV in *BRCA1/2* [10, 11]. Tumour genetic testing is particularly important for HGSOC, as 23–43% of *BRCA1/2* PVs are somatic and would be missed by standard germline (i.e. blood) genetic testing [12–14]. In August 2018, Ontario became the first Canadian province to implement reflex *BRCA1/2* tumour genetic testing for all newly diagnosed HGOSC patients.

Reflex tumour genetic testing refers to tumour testing that is completed automatically as part of standard pathology review protocols. It can detect both somatic and germline PVs, although the type of PV (germline or somatic) can not be distinguished. Unlike other tumour genetic tests where PV may be unlikely to confer a hereditary cancer risk, additional germline testing is required when a *BRCA1/2* PV is identified via tumour genetic testing [15, 16]. By eliminating potential patient, clinician, resource, and other system barriers to access genetic testing, reflex tumour genetic testing has the potential to streamline patient care pathways, resulting in improved rates of genetic testing, decreased time to results, and ultimately improved patient outcomes through personalized cancer care [17, 18].

Reflex tumour genetic testing of *BRCA1/2* represents a major shift from the traditional germline genetic testing model for HGSOC patients, and the potential psychological impact for patients needs to be considered. Traditionally, HGOSC patients access germline genetic testing following a ‘pre-test’ appointment with a genetic counsellor where the benefits, limitations, and possible results of genetic testing are reviewed so an individual can make an informed decision about testing. Numerous alternative models of genetic service delivery have been implemented to increase patient access to germline genetic testing [19]; ovarian cancer patients do not experience significant distress and are satisfied with models where germline genetic testing is ordered without prior genetic counselling [20–22]. While patient attitudes towards tumour genetic testing have been published [23], these studies have focused primarily on patients with advanced disease for the purpose of identifying therapeutic targets. To date, no studies have evaluated psychological outcomes following reflex tumour *BRCA1/2* genetic testing among newly diagnosed ovarian cancer patients.

The primary objective of this study was to measure the psychological impact of reflex *BRCA1/2* tumour genetic testing reported among newly diagnosed HGSOC patients. Secondary objectives were to 1) identify factors associated with patient reported psychological outcomes; 2) measure the level of hereditary breast/ovarian cancer knowledge among HGSOC patients; and 3) determine the level of satisfaction with reflex *BRCA1/2* tumour genetic testing reported among HGSOC patients. By reporting on the psychological impact of *BRCA1/2* tumour genetic testing for ovarian cancer in Ontario, we hope to inform the successful implementation of tumour genetic testing at other institutions and for additional tumour types.

## Methods

### Study design

A cross-sectional survey study of newly diagnosed HGSOC patients was conducted at two academic centers in Toronto, Ontario. Eligible participants were recruited from the University Health Network (UHN) from December 2019–August 2021 and from Sunnybrook Health Sciences Centre from December 2020 to August 2021.

### Study population & recruitment

Individuals with a new diagnosis of HGSOC were eligible to participate if they had: 1) pathology confirmed diagnosis of HGSOC; and 2) reflex *BRCA1/2* tumour genetic testing results reported in the past 3 months. Individuals were excluded if they had already received results of germline genetic testing for hereditary ovarian cancer risk or were unable to complete study surveys due to language barriers or cognitive capacity.

Eligible participants with an upcoming appointment were flagged for their gynecologic oncologist (one of the nine participating surgeons), who disclosed *BRCA1/2* tumour genetic testing results. Agreeable patients were approached by the study team to obtain informed consent. Consenting participants indicated their preferred method of completing the study survey (online, telephone or mail). Surveys were issued 1 week after disclosure of tumour genetic testing results; a reminder call or email was sent 1 and 2 weeks after the survey was provided.

### Measures

#### Clinical & sociodemographic data

Relevant clinical data (age at diagnosis, cancer stage, primary treatment, personal history of breast cancer, family history of breast/ovarian cancer, and tumour genetic testing results) were abstracted from the medical record. Survey items were used to obtain additional socio-demographic data (race/ethnicity, partner status, household income, level of education obtained) as well as participant recall of their tumour genetic testing results.

#### Life Orientation Test – Revised (LOT-R) scale

Dispositional optimism was evaluating using the LOT-R, which is a 10-item scale consisting of five-point Likert scale questions to measure dispositional optimism [24]. The six scored items include: 3 items for optimism and 3 items for pessimism (reversed scored), with the remaining 4 items considered filler questions. Total scores range from 0 to 24. There was no missing data among scored items in our dataset. Cronbach’s alpha was used to calculate the internal consistency of the scales in our cohort. The LOT-R scale demonstrated acceptable internal consistency (α = 0.79).

#### Impact of Event Scale (IES)

The IES was used to measure cancer-related distress. The scale consists of 15 four-point response items to measure subjective distress following a specific event or stressor [25]. In the current study, the event was defined as “having a diagnosis of ovarian cancer”. The scale is comprised of intrusion (7 items; score 0–35) and avoidance (8 items: score 0–40) subscales; total scores range from 0 to 75, with thresholds for subclinical (< 9), mild [9–25], moderate [26–43] and severe (44+) impact of an event [26]. One participant had missing data for 5/15 items and was excluded from analyses involving IES. No other missing item-level data was detected. Cronbach’s alpha was calculated with acceptable internal consistency for intrusion (α = 0.83), avoidance (α = 0.77), and total scores (α = 0.84).

#### Multidimensional Impact of Cancer Risk Assessment (MICRA) questionnaire

The MICRA questionnaire was developed to measure the psychological impact of genetic testing result disclosure [27]. It is comprised of 21 four-point response items, 19 of which are scored (total possible score:0–95). The MICRA questionnaire includes subscales for genetic testing-related distress (6 items: score 0–30), uncertainty (9 items: score 0–45), and positive experiences (4 items, reverse scored: 0–20). Positive experience items are reverse scored, meaning higher scores indicate a less positive experience. The MICRA questionnaire does not have established thresholds; however, higher scores indicate higher levels of psychological impact of genetic testing. Permission to use the MICRA questionnaire was obtained from the Functional Assessment of Chronic Illness Therapy (FACIT) Organization (FACIT.org). Per FACIT instructions, mean imputation was used to account for item-level missing data. Due to an initial error in survey development, one item of the MICRA questionnaire “Being uncertain about what my tumour genetic test result means about my cancer risk” (included in total and uncertainty scores), was missing from the survey of 20 participants. Analyses involving the MICRA questionnaire were run with and without this item and its inclusion did not significantly impact study findings. The total scale had an acceptable internal consistency (α = 0.86); internal consistencies were also calculated for distress (α = 0.85), uncertainty (α = 0.76) and positive experience (α =0.59) subscales. Due to the low internal consistency in our cohort, scores for the positive experience subscale are not reported.

#### Knowledge of hereditary breast/ovarian cancer

Participant knowledge of hereditary breast/ovarian cancer was assessed using a modified *BRCA* knowledge scale. Developed by the National Center for Human Genome Research, the *BRCA* knowledge scale consists of 11 true/false items measuring four aspects of hereditary cancer: the prevalence of *BRCA1/2* mutations, the patterns of inheritance, cancer risks associated with *BRCA1/2* mutations, and risk management options for women with *BRCA1/2* mutations [28]. Four true/false questions, developed in consultation with clinical experts, were added to measure knowledge of tumour versus germline genetic testing and potential treatment implications of tumour genetic testing results (Additional file 2). Knowledge scores were presented as the total percentage of correct responses; incomplete items were scored with a value of zero.

#### Satisfaction with reflex BRCA1/2 tumour testing

Participant reported satisfaction was assessed using a five-point Likert scale question.

### Data analysis

Descriptive statistics were used to provide a summary of study variables. Variables were reported using means and standard deviations or frequencies and percentages, for continuous and categorical variables, respectively. To address the primary objective of describing the psychological impact of tumour genetic testing, MICRA mean scores, along with standard deviations, were calculated for the total score and two subscales (distress, uncertainty).

Secondary objectives were addressed as follows: Univariable and multivariable analyses were undertaken to determine factors associated with all MICRA scores (total and subscale scores). MICRA scores were compared across levels of categorical variables using an independent t-test. Linear regression was used to measure the association of continuous variables with MICRA scores. Ordinary Least Square regression was used to undertake multivariable analysis, where included variables (dispositional optimism (total LOT-R score), age at diagnosis, having children (yes/no), positive tumour genetic testing results (yes/no), and cancer related distress (total IES score) were selected a priori based on literature review and consultation with clinicians. Primary treatment (surgery/neoadjuvant chemotherapy) was added to the multivariable models post-hoc given its highly significant association with multiple MICRA outcomes. Hierarchial regression was completed where cancer-related distress was included in a second regression block. A minimum of 60 participants were required for analysis as 10 participants per predictor variable is considered appropriate for regression models with more than six predictor variables [29]. Regression coefficients, 95% confidence intervals, the adjusted coefficient of determination, and *p* values were reported. Participant knowledge and satisfaction were reported using descriptive statistics.

Statistical analyses were completed using IBM SPSS Statistics for Windows, version 28 (Armonk NY, USA: IBM Corp) and statistical significance was reported using a two-tailed α = 0.05. Given the exploratory nature of this study, statistical corrections for multiple testing were not completed.

## Results

### Study participants

A total of 112 HGSOC patients met study inclusion criteria and were invited to participate. Of these, 84 (75.0%) consented and76 (80.5%) completed the study survey, for an overall response rate of 67.9%. The mean age at diagnosis was 62 years; most (88.2%) were diagnosed with stage III/IV disease and just over half (56.6%) were treated with primary surgery. Participant demographics are summarized in Table 1.

**Table 1:** Participant Demographics

	n (%)
**Study site**	
Princess Margaret	65 (85.5%)
Sunnybrook	11 (14.5%)
**Age at diagnosis**	
Mean (SD)	62.0 (10.7)
Minimum, Maximum	38, 84
**Ovarian cancer stage**	
I/II	9 (11.8%)
III/IV	67 (88.2%)
**Primary treatment**	
Neoadjuvant chemotherapy	33 (43.4%)
Primary surgery	43 (56.6%)
**Personal history breast cancer**	
Yes	6 (7.9%)
No	70 (92.1%)
**Family history breast or ovarian Cancer**	
Yes	34 (44.7%)
No	42 (55.3%)
**Previous genetic testing in the family**	
Yes	11 (14.7%)
No	64 (85.3%)
**Pre-test counselling prior to survey**	
Yes	31 (40.8%)
No	45 (59.2%)
**Tumour genetic test result**	
Positive (*BRCA1/2* pathogenic variant identified)	23 (30.3%)
Negative	53 (69.7%)
**Relationship Status**	
Partnered	43 (57.3%)
Not partnered	32 (42.7%)
Missing	1
**Children**	
Children	43 (56.6%)
No children	33 (43.4%)
**Education and training**	
Postsecondary education/training	61 (82.4%)
Secondary school or less	13 (17.6%)
Missing	2
**Household income**	
75 K or greater	24 (50.0%)
Less than 75 K	24 (50.0%)
Missing	28
**Race/Ethnicity**	
White	52 (71.2%)
Other	21 (28.8%)
Missing	3

Demographic information for the entire study cohort (*n* = 76)

### Tumour results

A PV in *BRCA1/2* was identified in 23 (30.3%) of HGSOC tumours. Thirteen (17.1%) individuals reported that they never received their tumour genetic testing results. Another 16 (21.1%) recalled receiving their tumour results but were unsure what their results were, 7 (43.8%) of whom had a PV in their tumour. Of 47 individuals who recalled a result, 2 (4.2%) reported their results incorrectly (1/13 with positive and 1/34 with negative results).

### Dispositional optimism

The mean LOT-R score for the total study cohort was 14.2 (SD = 5.2).

### Cancer-related distress

Intrusion, avoidance, and total cancer-related distress scores were calculated for all participants who completed the IES (*n* = 75). The mean intrusion and avoidance scores were 11.0 (SD = 7.6) and 15.0 (SD = 8.9), respectively. The mean total IES score was 26.0 (SD = 14.2); 39 (52.0%) individuals scored ≥26, indicating at least a moderate level of cancer-related distress and 9 (12.0%) scored ≥44 indicating a severe level of cancer-related distress.

### Psychological impact of genetic testing

The psychological impact of tumour genetic testing was measured for 63 individuals; the 13 individuals who did not receive their tumour genetic testing results were not asked to complete the MICRA questionnaire. The average total MICRA score was 26.8 (SD = 16.3), with subscales scores of 6.0 (SD = 6.8) for distress, and 13.3 (SD = 9.0) for uncertainty. In addition to scored items, the MICRA questionnaire includes 2 unscored items related to coping with cancer and 2 unscored items related to children (See additional file 1). Six (9.5%) individuals sometimes/always felt their tumour genetic testing results made it harder to cope with their cancer, and 36/61 (59.0%) sometimes/often felt results made it easier to cope with their cancer. Of 33 individuals who answered questions related to having children, 27 (81.8%) reported they were sometimes/often worried about the possibility of their children getting cancer and 22 (66.7%) sometimes/often felt guilty about the possibility of passing on a cancer risk to their children. Of these 22, 10 individuals had a positive tumour result, 5 of whom accurately reported this information.

Univariate analyses of factors associated with subscales and total score of the MICRA (Tables 2 and 3), with the following results:

**Table 2:** Univariable associations of categorical variables and MICRA scores

	**Distress**		**Uncertainty**		**Total**	
	Mean (SD)	***p***-value	Mean (SD)	***p***-value	Mean (SD)	***p***-value
**Partnered**		0.84		0.24		0.37
Yes	6.0 (6.8)		12.0 (9.6)		25.0 (16.3)	
No	5.6 (6.4)		14.8 (7.9)		28.7 (15.8)	
**Children**		0.10		**0.006**		**0.02**
Yes	7.4 (7.2)		16.2 (8.6)		31.4 (16.3)	
No	4.5 (6.1)		10.1 (8.5)		21.9 (15.0)	
**Post-secondary education**		0.17		0.80		0.64
Yes	6.2 (7.0)		13.0 (9.1)		26.9 (16.6)	
No	3.8 (4.3)		13.8 (8.7)		24.3 (13.7)	
**Household income**		0.40		0.09		0.29
< 75, 000 CAD	7.9 (7.5)		17.0 (10.0)		32.4 (18.0)	
≥ 75, 000 CAD	6.0 (6.5)		11.9 (8.6)		26.6 (16.5)	
**Race/Ethnicity**		0.75		0.37		0.70
White	6.1 (7.0)		12.5 (8.3)		26.1 (16.0)	
Other	5.4 (6.1)		14.9 (10.2)		27.9 (15.1)	
**Cancer stage**		0.89		0.73		0.71
I/II	6.8 (7.3)		14.3 (8.3)		28.8 (15.3)	
III/IV	5.9 (6.8)		13.1 (9.2)		26.6 (16.6)	
**Tumour results**		0.23		0.16		0.10
Positive	7.7 (7.7)		15.6 (9.1)		31.9 (17.4)	
Negative	5.3 (6.3)		12.2 (8.9)		24.6 (15.4)	
**Primary treatment**		**0.002**		**0.009**		**0.02**
Neoadjuvant chemotherapy	3.0 (4.8)		9.6 (7.5)		21.1 (12.8)	
Surgery	7.9 (7.2)		15.6 (9.2)		30.4 (17.4)	
**Personal history breast cancer**		**0.004**		0.63		0.35
Yes	2.0 (2.0)		11.3 (8.1)		20.3 (9.8)	
No	6.4 (6.9)		13.5 (9.1)		27.5 (16.7)	
**Family history breast/ovarian cancer**		0.13		0.46		0.17
Yes	7.4 (7.5)		14.2 (9.4)		29.9 (17.9)	
No	4.8 (5.9)		12.5 (8.7)		24.2 (14.4)	
**Previous genetic testing in family**		0.77		0.91		0.81
Yes	6.7 (7.3)		13.7 (9.5)		28.3 (17.7)	
No	6.0 (6.8)		13.4 (9.0)		27.0 (16.1)	
**Genetic counselling prior to survey**		0.45		0.56		0.21
Yes	5.3 (6.9)		12.5 (9.2)		24.0 (15.6)	
No	6.6 (6.7)		13.9 (8.9)		29.2 (16.7)	

Univariable results from the subset of participants who completed the MICRA questionnaire (*n* = 63)

**Table 3:** Univariable associations of continuous variables and MICRA scores

Variable	Distress		Uncertainty		Total Score	
	***B*** (95% CI)	***p***-value	***B*** (95% CI)	***p***-value	***B*** (95% CI)	***p***-value
**Age at diagnosis**	−0.13 (− 0.29, 0.03)	0.11	− 0.35 (− 0.55, − 0.15)	**< 0.001**	− 0.43 (− 0.81, − 0.05)	**0.03**
**Dispositional optimism**	− 0.36 (− 0.66, − 0.04)	**0.03**	−0.66 (− 1.05, − 0.26)	**0.001**	− 1.23 (− 1.94, − 0.52)	**< 0.001**
**Cancer-related distress**						
IES Intrusion	0.57 (0.41, 0.74)	**< 0.001**	0.79 (0.58, 1.00)	**< 0.001**	1.43 (1.05, 1.81)	**< 0.001**
IES Avoidance	0.18 (0.01, 0.37)	0.07	0.48 (0.25, 0.71)	**< 0.001**	0.59 (0.14, 1.04)	**0.01**
IES Total	0.25 (0.14, 0.35)	**< 0.001**	0.43 (0.32, 0.55)	**< 0.001**	0.68 (0.44, 0.92)	**< 0.001**
**Knowledge**	0.02 (− 0.14, 0.11)	0.78	−0.07 (− 0.23, 0.09)	0.40	− 0.06 (− 0.35, 0.23)	0.69

Univariable results from the subset of participants who completed the MICRA questionnaire (*n* = 63)

### Factors associated with MICRA distress subscale scores

Several factors were associated with higher MICRA distress scores, including treatment with primary surgery (*p* = 0.002) and higher total cancer-related distress (*p* < 0.001). Significantly lower MICRA distress scores were seen in individuals with a prior diagnosis of breast cancer (*p* = 0.004) and higher disposition optimism (*p* = 0.03).

### Factors associated with MICRA uncertainty subscale scores

Higher MICRA uncertainty scores were seen among individuals with children (*p* = 0.006), treatment with primary surgery (*p* = 0.009), and higher cancer-related distress (*p* < 0.001). Lower uncertainty scores were noted in those with an older age at diagnosis (*p* < 0.001) and higher dispositional optimism (*p* = 0.001).

### Factors associated with MICRA Total score

Having children (*p* = 0.02), treatment with primary surgery (*p* = 0.02), and higher cancer-related distress (*p* < 0.001) were associated with higher total MICRA scores. Older age at diagnosis (*p* = 0.03) and higher levels of dispositional optimism (*p* < 0.001) were associated with lower total MICRA scores.

### Multivariable model

A summary of multivariable models is presented in Table 4. In block 1 (excluding IES scores), treatment with primary surgery was associated with higher genetic testing-related distress (*p* = 0.04). Higher levels of dispositional optimism (*p* < 0.001) and increased age at diagnosis (*p* < 0.001) were associated with lower uncertainty scores, whereas having children was associated with higher uncertainty scores (*p* = 0.01). As for the overall psychological impact to tumour genetics testing, higher levels of dispositional optimism (*p* < 0.001) and increased age at diagnosis (*p* = 0.01) were both associated with lower total MICRA scores. With the addition of cancer-related distress (Block 2), higher cancer-related distress was associated with higher genetic testing-related distress (*p* = 0.002). Higher levels of dispositional optimism (*p* = 0.03) and increased age at diagnosis (*p* = 0.01) remained significantly associated with lower uncertainty scores. Having children (*p* = 0.04) and increased cancer-related distress (*p* < 0.001) were associated with higher uncertainty scores. Regarding the overall psychological impact of tumour genetics testing, increased dispositional optimism was associated with lower total MICRA scores (*p* = 0.03) and increased cancer related distress was associated with higher total MICRA scores (*p* < 0.001).

**Table 4:** Multiple linear regression models to identify variables associated with MICRA scores

	Distress		Uncertainty		Total Score	
	***B*** (95% CI)	***p***-value	***B*** (95% CI)	***p***-value	***B*** (95% CI)	***p***-value
**Block 1**						
Dispositional	−0.29 (− 0.60, − 0.02)	0.06	−0.65 (− 0.98, − 0.31)	**< 0.001**	− 1.21 (− 1.88, − 0.53)	**< 0.001**
optimism	− 0.10 (− 0.26, 0.06)	0.20	− 0.36 (− 0.53, − 0.18)	**< 0.001**	− 0.45 (− 0.79, − 0.10)	**0.01**
Age at diagnosis	2.32 (− 0.95, 5.59)	0.16	5.05 (1.44, 8.66)	**0.01**	7.04 (0.14, 14.22)	0.05
Children	0.98 (− 2.56, 4.52)	0.58	1.10 (− 2.81, 5.01)	0.57	4.26 (− 3.52, 12.04)	0.28
PV present in tumour	3.70 (0.22, 7.17)	**0.04**	2.70 (− 1.13, 6.54)	0.16	3.92 (− 3.71, 11.56)	0.31
Primary surgery	**R**^**2**^_**adj**_ = 0.172		**R**^**2**^_**adj**_ = 0.427		**R**^**2**^_**adj**_ = 0.308	
**Block 2**						
Dispositional	−0.09 (− 0.40, 0.22)	0.55	−0.35 (− 0.66, − 0.04)	**0.03**	− 0.73 (− 1.39, − 0.06)	**0.03**
optimism	− 0.005 (− 0.16, 0.15)	0.95	− 0.21 (− 0.37, − 0.05)	**0.01**	− 0.21 (− 0.55, 0.13)	0.21
Age at diagnosis	1.19 (− 1.91, 4.29)	0.45	3.38 (− 0.23, 6.52)	**0.04**	4.33 (− 2.35, 11.01)	0.20
Children	2.16 (− 1.20, 5.51)	0.20	2.85 (0.55, 6.27)	0.10	7.09 (1.14, 14.32)	0.05
PV present in tumour	2.86 (− 0.40, 6.11)	0.08	1.46 (− 1.85, 4.76)	0.38	1.91 (− 5.11, 8.92)	0.59
Primary surgery	0.21 (0.08, 0.33)	**0.002**	0.31 (0.18, 0.44)	**< 0.001**	0.50 (0.22, 0.77)	**< 0.001**
Cancer-related distress	**R**^**2**^_**adj**_ = 0.293		**R**^**2**^_**adj**_ = 0.587		**R**^**2**^_**adj**_ = 0.431	

*PV* pathogenic or likely pathogenic variant

Multiple linear regression model for the subset of participants who completed the MICRA questionnaire (*n* = 63)

### Knowledge of hereditary breast/ovarian cancer

Average knowledge score among our participants was 66.8%. Scores were similar for *BRCA* knowledge items (65.8% correct) and questions about tumour testing (69.7% correct). Individual items and the percentage of correct responses are presented in Additional file 2.

### Satisfaction with tumour testing

Seventy-three participants ranked their level of satisfaction; 61 (83.5%) reported being satisfied/highly satisfied with having reflex *BRCA1/2* tumour genetic testing completed as part of their cancer care. Among 23 patients with a positive tumour result, 18 (78.2%) reported they were satisfied/highly satisfied.

## Discussion

This is the first study to investigate the psychological impact of reflex *BRCA1/2* genetic testing among HGSOC patients. Partients’ reported level of cancer-related distress had the most significant association with levels of genetic testing-related distress, uncertainty, and the overall psychological impact of tumour genetic testing. Higher optimism scores and increased age at diagnosis reduced the psychological impact of reflex tumour genetic testing in our cohort; however, when controlling for cancer-related distress, these protective effects decreased. Despite ensuring that all patients had tumour results disclosed by their gynecologic oncologist, many could not accurately recall their results; nevertheless, most participants were satisfied with having reflex *BRCA1/2* tumour genetic testing completed as part of their cancer care.

Previous research suggests that ovarian cancer patients have favourable attitudes towards germline genetic testing [30–32], and germline genetic testing is not typically disruptive in the context of an ovarian cancer diagnosis [20, 21]. Given the benefits of targeted therapy with a PARPi among HGSOC patients with a *BRCA1/2* PV, it is possible that receiving positive *BRCA1/2* tumour genetic testing result may be perceived as ‘good’ news. The mean psychological impact of tumour genetic testing among ovarian cancer patients in this study was measured as 26.8 via the MICRA questionnaire. Though there is no cut-off to define a “high” MICRA score, this score is higher than recently published Norwegian (mean score = 17.7) [33, 34] and American (mean score = 20) [33, 34] cohorts of ovarian cancer patients who received germline genetic testing results. It should be noted that individuals in the Norweigan and Amerian cohorts completed the MICRA questionnaire an average of 4.6 and 5.4 years after their ovarian cancer diagnosis and 31.0 and 12.6 months after receiving their genetic test results, respectively. Though the longer time from diagnosis and receipt of results may have reduced the psychological impact of genetic testing, the MICRA score in our cohort was also higher than that recently reported in a Canadian cohort of breast/ovarian cancer patients whose germline testing was arranged directly by their oncologist, during a regularly scheduled oncology visit (mean score = 20) [35]. While the authors of this study do not report the time from diagnosis to testing, the results of this study may provide a more direct comparison to ours as oncologist-ordered genetic testing is often completed early in a patient’s cancer journey to determine appropriate therapies and the MICRA tool was administered 1 month following results disclosure.

When evaluating the psychological impact of genetic testing, results must be considered in the context of an individual’s personal experience. Baseline distress has been cited as the biggest risk factor for immediate psychological distress following germline genetic testing [36], and reported levels of cancer-related distress were highly associated with levels of psychological impact of tumour genetic testing in our cohort (*p* < 0.001). Many ovarian cancer patients experience distress, particularly after initial diagnosis [37, 38]. Our cohort consisted of individuals with a recent ovarian cancer diagnosis, over half of whom reported significant cancer-related distress. The mean level of cancer-related distress in our cohort (total IES score of 26.0) was higher than reported in another published cohort of ovarian cancer patients [38]. These findings suggest that the heighted psychological impact of tumour genetic testing in our cohort may be a result of a recent cancer diagnosis. In contrast, dispositional optimism was associated with a lower psychological impact of tumour genetic testing in our cohort (*p* < 0.001). Individuals with higher dispositional optimism are expected to have a ‘glass half full’ outlook and may focus on the benefits of genetic information, such as identifying targeted therapies. Yet, the average level of dispositional optimisim reported in our cohort (total LOT-R score of 14.2) was lower than published scores among ovarian cancer patients [39] and individuals with a new cancer diagnosis [40]; thus, low levels of dispositional optimism may also contribute to the relatively high psychological impact of tumour genetic testing reported in our cohort. Another possible explanation is that the MICRA questionnaire is designed to evaluate the impact of receiving germline genetic information. Given differences in the information, purpose, and potential consequences of germline versus tumour genetic testing, new scales may be required to accurately evaluate the psychological effect of tumour genetic testing.

Several additional factors were associated with the psychological impact of reflex *BRCA1/2* tumour genetic testing in our cohort. Consistent with published literature of germline genetic testing [20, 22, 33, 34], younger age at diagnosis was associated with an increased psychological impact of tumour genetic testing (*p* = 0.03). This may be due to the increased levels of psychological distress experienced by younger individuals following a diagnosis of ovarian cancer [38]. Younger individuals may also have young children at home to consider; 81.8% of parents in our study cohort worried about the possibility of their children getting cancer, and having children was also associated with an increased psychological impact of tumour genetic testing (*p* = 0.02). Of interest, a personal history of breast cancer was associated with significantly lower genetic testing related distress (*p* = 0.004) in our cohort. For these individuals, genetic testing may be of minimal concern when facing a second cancer diagnosis; however, only six individuals has a previous diagnosis of breast cancer completed the full study survey and definitive conclusions cannot be made on such small numbers. Finally, treatment with primary surgery, as opposed to neoadjuvant chemotherapy, was associated with a higher psychological impact of tumour genetic testing in our study (*p* = 0.02). Since reflex *BRCA1/2* tumour genetic testing is typically arranged on a surgical specimen, this may be a function of decreased time from diagnosis to receipt of tumour genetic testing results. Individuals who were treated with primary surgery may also be starting adjuvant chemotherapy treatments at the time of result disclosure, which may contribute to increased distress. Additional studies are needed to better interpret these findings.

Previous research suggests that positive genetic test results are associated with a higher psychological impact of genetic testing [33, 34, 41]. In contrast, receiving a positive tumour genetic testing result was not significantly associated with any MICRA scores (genetic testing related distress, uncertainty, or overall psychological impact) in our cohort. The finding further supports the idea that the relatively high MICRA scores may be a result of factors outside of tumour genetic testing, such as having a recent cancer diagnosis. Importantly, almost half (11/23) of individuals with *BRCA1/2* PV in their tumour could not correctly recall their genetic testing result, which likely translates to an inaccurate evaluation of the psychological effect of receiving a positive *BRCA1/2* tumour genetic testing result.

Overall, a large proportion (40.8%) of HGSOC patients in our study could not accurately recall their tumour genetic testing results. Likewise, in a study of individuals with advanced-stage solid tumour malignancy, 25% could not recall disclosure of their tumour genetic test results, despite documentation that such a discussion had occurred [42]. It is possible that cancer patients have a hard time interpreting, and therefore remembering, their tumour genetic test, particularly in reflex testing context, where individuals are not aware that tumour genetic testing will be completed and high levels of distress may impact their ability to integrate genetic information. Our results suggest that patients may benefit from post-test genetic counselling and/or educational resources following *BRCA1/2* tumour testing in order to better understand and recall their results. Although 40.8% of participants in our study had met with a genetic counsellor prior to completing the study survey, it is unknown whether *BRCA1/2* tumour results were available or discussed during their appointment.

The difference between tumour and germline genetic testing, including the types of information gleaned, is a complicated concept for many patients to understand. Previous literature states that cancer patients have moderate to poor knowledge of tumour genetic testing and most are unaware of the difference between tumour and germline genetic testing [23]. Most individuals in this study reported tumour genetic testing may help their doctors direct their treatment, but many thought that *BRCA1/2* PV variants seen in tumour tissue were always inherited from a parent. In a qualitative study of individuals with advanced cancer who consented to a large tumour genetic testing program in Australia, participants acknowledged their level of knowledge was poor, but stated this did not impact their decision have tumour genetic testing [43]. Combined the finding that 83.5% of HGSOC in our study were satisfied/highly satisfied with having reflex *BRCA1/2* tumour genetic testing completed as part of their cancer care, available data suggest gaps in knowledge about tumour genetic testing may be of minimal concern for cancer patients. Nevertheless, additional education or post-test genetic counselling may be of benefit to ensure that cancer patients understand their results, particularly when testing genes like *BRCA1/2* where many PV are germline in orgin and confer a hereditary cancer risk.

The results of this study should be considered in context of its strengths and limitations. The provincial implementation of reflex tumour genetic testing for all HGSOC patients in the province of Ontario allowed us to evaluate the ‘real world’ experience of a diverse cohort of individuals. At the same time, the pragmatic approach of this study introduced variability in the information provided, as tumour genetic testing were disclosed by one of nine gynecologic oncologists. Such variation cannot be accounted for without a much larger sample size. Similarly, a larger sample size would allow for the inclusion of additional potentially relevant variables in our multivariable model and to identify factors which may have small effects on psychological outcomes. Our study is also limited in its cross-sectional design, and future studies, including longitudinal analyses, qualitative studies and direct comparisons of the psychological response to tumour and germline genetic testing, may provide better insight into the patient experience and areas where additional support is required. Using the MICRA questionnaire, which is designed to measure the psychological impact of germline genetic testing, may have given an inaccurate representation of experiences with tumour genetic testing. Additionally, the MICRA positive experience subscale had poor internal consistency in our cohort and we were unable to conduct reliable analyses for this measure. Further evaluation of the MICRA questionnaire in a tumour genetic testing context is required to determine whether the development of new measures is required. Finally, this study was conducted during the global COVID-19 pandemic and results should be interpreted with the knowledge that ovarian cancer patients experienced high levels of distress during this time [44].

## Conclusions

This study provides a first-look into the experience of newly diagnosed HGSOC receiving reflex *BRCA1/2* tumour genetic testing. The psychological impact of reflex tumour genetic testing in our cohort was higher than published data of ovarian cancer patients undergoing germline genetic testing; however, this may be a function of receiving a recent cancer diagnosis. Increased dispositional optimism provided a protective effect, but higher levels of cancer-related distress had the most significant association with psychological outcomes following tumour genetic testing. Knowledge scores and poor recall of tumour genetic testing results suggest that HGSOC patients may benefit from educational resources and/or post-test genetic counselling following receipt of *BRCA1/2* tumour genetic test results. Additional research, including qualitative studies, are needed to better understand the patient experience of reflex *BRCA1/2* tumour genetic testing.

## Data Availability

The datasets generated and analysed during the current study are available from the corresponding author on reasonable request.

## Abbreviations

HGSOC: High-grade serous ovarian cancer
PARPi: Poly(ADP-ribose) polymerase inhibitors
MICRA: Multidimensional Impact of Cancer Risk Assessment
IES: Impact of Event Scale
LOT-R: Life Orientation Test-Revised
PV: Pathogenic variant
